# Evidence of patients’ challenges and barriers related to usage of Implanon®: scoping review protocol

**DOI:** 10.1186/s13643-018-0827-1

**Published:** 2018-10-12

**Authors:** Shimona Prosad, Tivani P Mashamba-Thompson, Elizabeth Ojewole

**Affiliations:** 1 Discipline of Pharmaceutical Sciences, School of Health Sciences, College of Health Sciences, University of KwaZulu-Natal, Westville Campus, P B X54001, Durban, 4000 South Africa; 2 TPMT- Discipline of Public Health Medicine, School of Nursing and Public Health, College of Health Sciences, University of KwaZulu-Natal, Howard Campus, P B X54001, Durban, 4000 South Africa

**Keywords:** Etonogestrel implant, Implanon®, Usage, Contraceptive, Barriers, Challenges

## Abstract

**Background:**

According to the United Nations Trends in Contraceptive Use 2015 report, at least one in ten married or in-union women in most regions of the world has an unmet need for family planning. Family Planning 2020 reports an estimate of almost 134 million married or in-union women of reproductive age who have an unmet need for modern methods of contraception in 2016 in participating countries. Family planning has therefore been highlighted as a global unmet need. Initiatives such as Family Planning 2020 aim to promote contraceptive use through Implanon® contraceptive implant. Implanon® has been reported to be a highly effective form of contraception. However, poor outcomes from users of the Implanon® have been reported in recent studies. The main objective of this review is to map the literature for the evidence on usage of Implanon® in order to reveal challenges and barriers.

**Methods and analysis:**

A scoping review searching evidence on Implanon® use will be conducted. Relevant studies will be identified from 1998 to present. The following databases: PubMed, MEDLINE, EBSCOhost, Google Scholar and Cochrane library will be searched for peer-reviewed literature. We will also search for grey literature in this study area. The eligibility criteria will guide the study selection. A data charting table will be designed to extract information from the literature. The results of this study will be reported by use of the Preferred Reporting Items for Systematic Reviews and Meta-Analyses (PRISMA). Data will be analysed using thematic analysis and the NVIVO software version 10. The mixed method appraisal tool (MMAT) will be used to assess the quality of included studies.

**Discussion:**

We anticipate finding relevant studies on the use of Implanon®. Evidence gathered from included studies will help us identify gaps in research and help guide future research on Implanon® usage. This information can also help guide implementers and users on challenges and barriers related to use of Implanon®.

**Systematic review registration:**

PROSPERO CRD42017072926.

## Background

Implanon® is a subdermal contraceptive implant that is classified as a long-acting reversible contraceptive. It was initially released in 1998 in Indonesia. Since 1998, more than 3.3 million implants have been dispensed globally [1]. The 2015 United Nations Trends in Contraceptive Use Worldwide report states that more than one in three married or in-union women globally use long-acting or permanent methods namely sterilisation, intrauterine device, and implants [2]. According to the FP2020 Momentum at the Midpoint 2015–2016 report, injectables and implants are the fastest growing methods globally [3]. Implanon® has been reported as a highly efficacious contraceptive [4–8]. However, problems such as adverse effects, early discontinuation of the product, and contraceptive failure have been reported in a variety of studies published globally [1, 8–12].

Despite the reported failures related to Implanon® use, the use of Implanon® is still being promoted globally. Initiatives such as Family Planning 2020 aim to address an unmet need for family planning services through distribution of modern contraceptives like Implanon® [3]. Their goal is to enable 120 million more females to use contraceptives by 2020 [3]. According to the FP2020 2015–2016 progress report, there were 30.2 million additional users of modern contraception compared to 2012 [3]. This movement will help to achieve the sustainable development goals (SDG), which is aimed at preventing unintended pregnancy and reduce adolescent childbearing through universal access to sexual and reproductive healthcare services [13]. This will also address the SDG, which focuses on gender equality and female empowerment and also aims to ensure universal access to sexual and reproductive health and reproductive rights [13].

Family planning has been highlighted as a global unmet need [2, 3, 13]. Implanon® has potentiation to address this unmet need by improving birth control and preventing unwanted pregnancies. The Pearl Index scores reported for Implanon® are similar to other long-acting reversible contraception as well as similar to sterilisation [4]. Implanon® is convenient, cost-effective, and highly efficacious compared to other contraceptives [14, 15]. Return to fertility is quick with Implanon®, and it can be safely used while breastfeeding [4, 7, 14]. It can also be used by women who cannot tolerate estrogen [4]. However, the challenges and barriers related to usage of Implanon® are not well known. The scoping review is aimed at mapping evidence on challenges and barriers linked to usage of this product since its introduction to the market.

It is anticipated that the results of this study will provide information on challenges and barriers related to Implanon® usage. The study findings will also guide future research as well as inform the policymakers and users of Implanon®.

The objectives of the scoping review are as follows:

- To review research reports on barriers and challenges of usage of Implanon®
- To review research reports on Implanon® users’ experiences
- To review research reports on Implanon®’s adverse effects
- To review research reports on discontinuation rate of Implanon® and reasons for discontinuation
- To review research reports on Implanon®’s failure rate

## Methodology

### Scoping review

We will conduct a scoping review with guidance from Arksey and O′ Malley’s scoping review framework [16, 17]. The adapted framework that will be used comprises of the following five stages:

Step 1: Identifying the research question
Step 2: Identifying relevant studies
Step 3: Study selection
Step 4: Charting the data
Step 5: Collating, summarising and reporting the results

#### Identifying the research question

The main research question is what evidence is available on the barriers and challenges of etonogestrel implant (Implanon®) usage? The secondary research questions are as follows:

1. What are patients’ experiences of Implanon® usage?
2. What are the adverse effects of Implanon®?
3. What is the discontinuation rate of Implanon® and the reasons for discontinuation?
4. What is the failure rate of Implanon® usage?

### Eligibility of research question

The Population Intervention Comparison Outcomes (PICO) framework will be used to determine the eligibility of the research question. PICO is used to break down clinical questions into searchable keywords [18]. Table 1 shows the PICO framework for the research question.

**Table 1:** PICO table to determine eligibility of research question

Criteria	Determinants
Population	Females who used or are using Implanon® as a contraceptive option
Intervention	Usage of Implanon®
Comparison	Absence of usage of Implanon®
Outcome	Main outcome: barriers and challenges related to Implanon® usage Secondary outcomes: patients’ experiences of Implanon® usage, adverse effects related to Implanon® usage, discontinuation rate of Implanon® use and reasons for discontinuation, failure rate of Implanon®

#### Identifying relevant studies

An electronic search will be conducted using the following databases: PubMed, EBSCOhost (Academic Search Complete, MEDLINE and CINAHL), Google Scholar and Cochrane library. Websites such as governmental websites, World Health Organisation and online newspaper sources will be searched for reports and articles related to the research question. We will also conduct a hand search of reference list of the included studies. Primary and secondary research studies that address the research question will be included. Studies will not be limited by method design or country. Grey literature will also be included. The Boolean search terms will include the following: females and use of Implanon® or barriers or challenges or experiences or adverse effects or discontinuation rate or reasons for discontinuation or failure rate.

#### Study selection

The eligibility criteria will be used to select appropriate studies.

## Eligibility criteria

### Inclusion criteria

- Articles reporting evidence of females who used or are using Implanon® as a contraceptive option
- Articles reporting evidence of barriers and challenges related to Implanon® usage
- Articles reporting evidence of Implanon® use
- Articles reporting evidence of patients’ experiences of Implanon® use
- Articles reporting evidence of Implanon® adverse effects
- Articles reporting evidence of discontinuation of Implanon® use
- Articles reporting evidence of reasons for discontinuation of Implanon®
- Articles reporting evidence of failure of Implanon® usage
- Articles published from 1998 to present
- All method designs for appropriate studies included

### Exclusion criteria

- Articles that do not report evidence of any experiences of Implanon® users
- Articles published before 1998
- Articles that report only on contraceptives other than Implanon®
- Articles that report health practitioners’ experiences with Implanon®

**Fig. 1:**
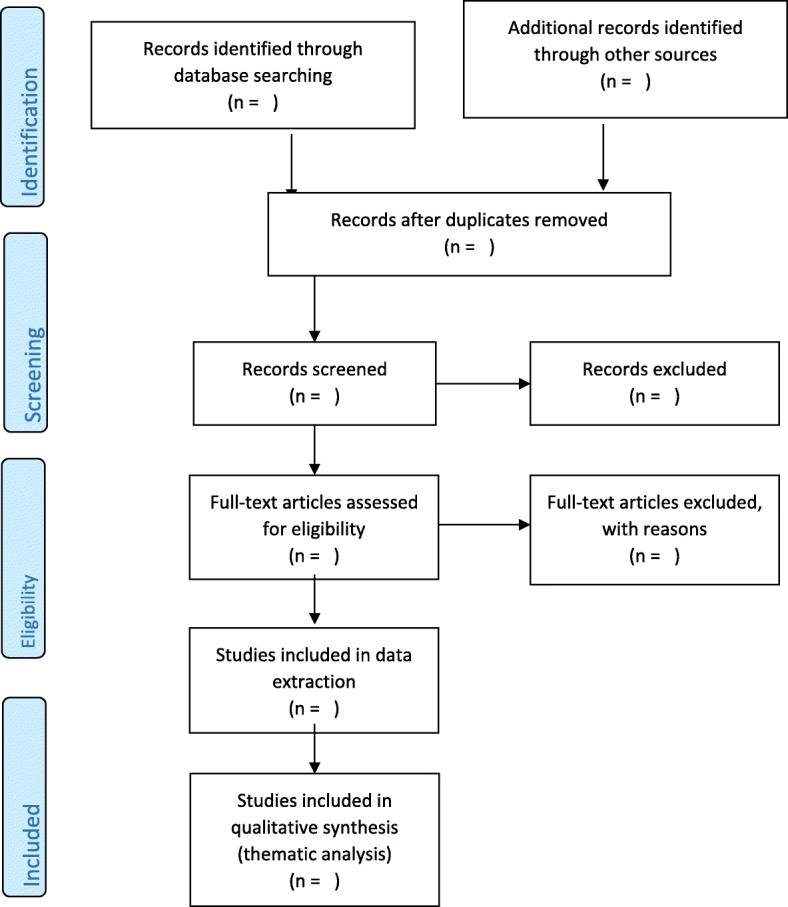
Study selection process

The methodology will be piloted to assess the appropriateness of the study design. Articles will be searched using the selected databases. The initial title screening will be done by the principal investigator. Included studies at title screening stage will be exported to a library on Endnote reference manager for abstract and full-article screening. Abstract and full-article screening will be guided by the eligibility criteria. The endnote library will be shared with a second reviewer for abstract and full-article screening. Any discrepancies in the results of abstract screening will be resolved through a discussion until consensus is reached. Discrepancies in full-article screening results will be resolved by a third screener. A PRISMA chart will be used to report the screening results (see Fig. 1). Authors of studies will be contacted to access missing studies. The University of KwaZulu-Natal library service will also be used to access articles that are not available online as full articles. Full articles will be requested from the author if articles are unavailable from the databases. We conducted a pilot search using our keywords, and database results are attached in Table 3 in the Appendix.

#### Data charting

A data charting table will be designed and used to extract data from included studies. A draft of the data charting form is depicted in Table 2.

**Table 2:** Data charting form

Author and date	
Title	
Reference	
Research question	
Aims and objectives	
Summary of the study results	
Sample size	
Age	
Marital status	
Setting	
Recruitment method	
Sampling method	
Study design	
Data collection method	
Data analysis	
Intervention	
Outcomes	
Relevant findings	
Conclusion	
Comments	

#### Collating, summarising and reporting of results

The data extracted from articles will be analysed using thematic analysis. The themes are derived from our study outcomes. These include the following: barriers and challenges related to Implanon® usage, patients’ experiences of Implanon® usage, adverse effects of Implanon® usage, discontinuation rate of Implanon®, reasons for discontinuation of Implanon®, and failure rates of Implanon®. Emerging themes will also be reported. NVIVO software version 10 will be used to code the data according to themes [19].

### Synthesis

The identified themes will be analysed, and their relationship to the research questions will be assessed. The analysis of findings will be discussed in relation to the study aim and objectives.

### Quality appraisal

Mixed Methods Appraisal Tool (MMAT) version 2011 will be used to appraise identified studies [20]. This tool allows one to assess the quality and appropriateness of the studies. A quality score will be generated using MMAT. We will score qualitative and quantitative studies by dividing the number of criteria met by four and presenting the score using *, **, ***, and **** descriptors. Scores will vary from 25% (*)—one criterion met—to 100% (****)—all criteria met. With regard to mixed methods research studies, the overall quality of a combination cannot exceed the quality of its weakest component therefore the overall quality score is the lowest score of the study components. The score is 25% (*) when QUAL = 1 or QUAN = 1 or MM = 0; it is 50% (**) when QUAL = 2 or QUAN = 2 or MM = 1; it is 75% (***) when QUAL = 3 or QUAN = 3 or MM = 2; and it is 100% (****) when QUAL = 4 and QUAN = 4 and MM = 3 (QUAL being the score of the qualitative component, QUAN the score of the quantitative component, and MM the score of the mixed methods component) [20]. Systematic reviews will be analysed under observational studies.

## Discussion

The scoping review will be conducted to map the existing peer-reviewed literature for evidence on challenges and barriers related to usage of Implanon®. Studies on problems with the use of Implanon® have been reported in most countries [1, 5, 9, 10, 21]. However, there is a paucity of literature on the evidence regarding patients’ challenges and barriers related to Implanon®. It is important to investigate the trends and extent of these problems, globally. There is a need to consolidate and evaluate this information to guide future practice and policy regarding Implanon®.

This study only focuses on the contraceptive implant Implanon® and not on other contraceptive options. Evidence from 1998 onwards will be screened. Implanon® was put on the market in 1998. Therefore, this study will map literature evidence on the usage of the product since its introduction on the market. Aspects such as experiences of health practitioners with Implanon® are not of interest to this study. The focus of the study is based on user experience with reference to pharmacovigilance of Implanon®. The perspective of the user is essential in pharmacovigilance reporting [22].

The findings from this review may be of interest to healthcare practitioners in terms of improving the provision of Implanon®. The review will also inform policy makers and may influence policy and guidelines related to the use of Implanon®. Researchers may also be interested in filling the gaps exposed through the review.

## Appendix

**Table 3:** Results of pilot search

Keyword search	Date of search	Search engine used	Number of publications retrieved (results)
((((((((“female”[MeSH Terms] OR “female”[All Fields] OR “females”[All Fields]) AND (“etonogestrel”[Supplementary Concept] OR “etonogestrel”[All Fields] OR “Implanon®” “[All Fields]))” OR Barriers[All Fields]) OR (“Plan Parent Chall”[Journal] OR “challenges”[All Fields])) OR Experiences[All Fields]) OR (“adverse effects”[Subheading] OR (“adverse”[All Fields] AND “effects”[All Fields]) OR “adverse effects”[All Fields])) OR (Discontinuation[All Fields] AND (“J Rehabil Assist Technol Eng”[Journal] OR “rate”[All Fields]))) OR (Reasons[All Fields] AND discontinuation[All Fields])) OR (Failure[All Fields] AND (“J Rehabil Assist Technol Eng”[Journal] OR “rate”[All Fields]))	25 July 2017	PubMed	2,583,613

## Abbreviations

MMAT: Mixed Methods Appraisal Tool
PICO: Population Intervention Comparison Outcomes
PRISMA/-P: Preferred Reporting Systematic Review and Meta-Analysis Protocols
SDG: Sustainable development goals
